# Neuro-Genomic Mapping of Cardiac Neurons with Systemic Analysis Reveals Cognitive and Neurodevelopmental Impacts in Congenital Heart Disease

**DOI:** 10.3390/life15091400

**Published:** 2025-09-04

**Authors:** Abhimanyu Thakur, Raj Kishore

**Affiliations:** 1 Aging and Cardiovascular Discovery Center, Lewis Katz School of Medicine, Temple University, Philadelphia, PA 19140, USA; 2 Department of Cardiovascular Sciences, Lewis Katz School of Medicine, Temple University, Philadelphia, PA 19140, USA

**Keywords:** congenital heart disease, neuro-innervation, cardiac neuron, exosome crosstalk, cognition, neurodevelopment

## Abstract

Congenital heart disease (CHD) is associated with neurodevelopmental and cognitive impairments, but the underlying molecular mechanisms remain unclear. This study investigated cardiac neuronal genomics in CHD using single-nucleus RNA-sequencing data (GSE203274) from 157,273 cardiac nuclei of healthy donors and patients with hypoplastic left heart syndrome (HLHS), Tetralogy of Fallot (TOF), dilated (DCM), and hypertrophic (HCM) cardiomyopathies. The Uniform Manifold Approximation and Projection (UMAP) clustering identified major cardiac cell types, revealing neuron-specific transcriptional programmes. Neuronal populations showed enriched expression of neurodevelopmental disorder-linked genes (*NRXN3*, *CADM2*, *ZNF536*) and synaptic signalling pathways. CHD cardiac neurons exhibited upregulated markers of cognitive dysfunction (*APP*, *SNCA*, *BDNF*) and neurodevelopment regulators (*DNMT1*, *HCFC1*) across subtypes. Cardiomyocyte troponin elevation correlated with neuronal exosome receptor expression (*TLR2*, *LRP1*), suggesting intercellular communication. Gene ontology analysis highlighted overlaps between cardiovascular disease pathways and neurodevelopmental disorder signatures in CHD neurons. These findings provide the first neuro-genomic map of cardiac neurons in CHD, linking cardiac pathology to neural outcomes through transcriptional dysregulation. Further, the systemic analysis of clinical findings in CHD further supports the risk of neurodevelopmental impacts. In summary, this study identifies transcriptional dysregulation within cardiac neurons in CHD and, together with a systemic analysis of clinical data, provides molecular evidence linking cardiac pathology to neurodevelopmental and cognitive impairments.

## 1. Introduction

Congenital heart disease (CHD), affecting nearly 1% of live births globally, has transformed from a fatal pediatric condition to a chronic multisystem disorder with growing recognition of neurodevelopmental sequelae [1,2,3]. While surgical advances enable >90% survival to adulthood, 30–40% of survivors exhibit cognitive impairments ranging from executive dysfunction to autism spectrum disorders, with fourfold increased psychiatric morbidity compared to the general population [4]. This neurological burden persists despite optimal cardiac care, suggesting intrinsic biological links between cardiac maldevelopment and neural pathogenesis that extend beyond perioperative factors [3].

Emerging evidence implicates shared genetic underpinnings in CHD and neurodevelopmental disorders (NDDs), with copy number variants at 1q21.1, 16p13.1-11, and 8p23.1 loci disrupting both cardio-genesis and cortical development [2,3]. Critical pathways include Wnt signalling regulators like HCFC1, epigenetic modifiers such as DNMT3A, and synaptic plasticity genes including NRXN3—all implicated in CHD-associated NDDs through population genomics [4]. These findings align with single-cell studies demonstrating that cardiac neuronal populations express neurodevelopmental transcription factors during embryogenesis, suggesting persistent molecular crosstalk [5]. However, the postnatal persistence of such programmes in CHD hearts remains unexplored, creating a critical knowledge gap in understanding cardio-cerebral connections.

Recent advances in single-nucleus RNA sequencing (snRNA-seq) have revolutionized our capacity to decode cellular heterogeneity in cardiovascular tissues [6,7]. Spatial transcriptomics further enable mapping of intercellular communication networks disrupted in disease states. Applying these tools to CHD, preliminary studies reveal cardiomyocyte-specific dysregulation of hypertrophy-related genes and fibroblast activation pathways [6], while developmental analyses identify Hand2-dependent transcriptional networks governing cardiac progenitor fate [7]. Nevertheless, neuronal components within cardiac tissue—long considered limited to autonomic regulation—have not been systematically characterized in CHD despite their potential role in mediating systemic inflammation and neurohumoral signalling.

The cardiomyocyte–neuronal axis may hold particular significance, given emerging links between cardiac troponin elevation and cognitive decline through putative exosome-mediated signalling. Animal models demonstrate that cardiomyocyte-derived exosomes carrying troponin complexes activate neuronal Toll-like receptors, inducing synaptic dysfunction through NF-κB pathways [4]. Human studies further correlate CHD-associated neurodevelopmental impairments with altered cerebral blood flow patterns and neurovascular coupling, suggesting that circulating cardiac factors modulate central nervous system homeostasis [2]. Reports have shown that early developmental delays in infants with CHD predict increased long-term neurodevelopmental risk; however, normal early development does not preclude later deficits, as many children develop impairments by school age despite typical infancy milestones [8,9,10]. However, the molecular mediators of this heart–brain crosstalk remain undefined, particularly within CHD-specific contexts.

In this study, we analyzed integrative single-nucleus RNA sequencing of 157,273 cardiac nuclei from donors and CHD patients, including HLHS and Tetralogy of Fallot (TOF), two common forms of cyanotic CHD lesions, as well as dilated (DCM) and hypertrophic (HCM) cardiomyopathies (Gene Expression Omnibus, GEO accession: GSE203274) [1], to examine whether the CHD hearts harbour aberrant neuronal transcriptional programmes linking cardiac pathology to neural comorbidities through persistent expression of neurodevelopmental regulators, cardiomyocyte–neuronal crosstalk via exosome signalling, and shared genomic architecture between cardiovascular and neurodevelopmental pathways. Subsequently, by leveraging the integration of single-cell genomics with clinical correlation analyses, this work paves the way for targeted therapeutic strategies against CHD-associated neurocognitive impairments.

## 2. Materials and Methods

### 2.1. Single-Nucleus RNA-Seq Dataset

The single-nucleus RNA sequencing dataset (GEO Accession: GSE203274) [1] was obtained from the public database accessible at Gene Expression Omnibus (GEO) (accessed on 24 January 2025). This dataset consists of 157,273 nuclei from donors and CHD patients, including HLHS and TOF, two common forms of cyanotic CHD lesions, as well as DCM and HCM.

### 2.2. Single-Nucleus RNA-Seq Data Analysis

The single-nucleus RNA-seq datasets were analyzed with the statistical programming language R (v. 4.4.2). The following packages were used for loading, saving, and manipulating data, as well as data integration, analysis, and generating plots: Seurat (v 4.3.0), tidyverse (v 1.3.2), dplyr (v 1.1.0), patchwork (v 1.1.2), stringr (v 1.5.0), Harmony (v 0.1.1), org.Hs.eg.db (v 3.16.0), and SingleR (v 2.0.0). The read count matrix was read into R, which subsequently created the Seurat object with the CreateSeuratObject () function. To remove the batch effect, all samples were integrated iteratively. Uniform manifold approximation projections (UMAPs) were then calculated using runUMAP(), respectively, using the first 20 dimensions. The analysis results were visualized using the FeaturePlot(), VlnPlot(), and DotPlot() functions. The single-cell portal (available at https://singlecell.broadinstitute.org/ (accessed on 24 January 2025)) was also utilized.

We performed differential gene expression analysis for each cell type labelled by the original study, comparing patient samples with control samples. The testing method was the Wilcoxon rank sum test. The Benjamin–Hochberg test was used for multiple testing corrections. The adjusted *p*-value cut-off was 0.05. Enrichment analysis was conducted using the function “enriched ()” in the R package “clusterProfiler” (version 4.16.0).

### 2.3. Gene Set Enrichment Analysis (GSEA)

GSEA was conducted using ShinyGO v0.82 to examine biological processes, cellular components, and molecular functions in cardiac neurons of CHD. Parameters included FDR *q*-value ≤ 0.25 and |Normalized Enrichment Score (NES)| ≥ 1.5. Results were visualized through GSEA-based dot plots and network graphs, highlighting enriched pathways across disease conditions. The network graph revealed associations between cardiovascular phenotypes (nodes 1–2) and neurodevelopmental disorders (nodes 3–5), with edges weighted by gene overlap similarity.

### 2.4. Systemic Analysis

Literature Search Strategy: A comprehensive literature search was conducted to identify studies evaluating the association between congenital heart disease (CHD) and neurodevelopmental disorders. Databases including PubMed, Scopus, and Web of Science were systematically searched using keywords such as “congenital heart disease,” “neurodevelopmental disorders,” “cognitive impairment,” and “odds ratio.” The search included articles published up to June 2025. Additional studies were identified by screening references of relevant reviews.

Study Selection Criteria: Eligible studies were required to meet the following criteria: (1) original research articles involving patients with CHD; (2) qualitative assessment of neurodevelopmental or cognitive outcomes; and (3) publication in peer-reviewed English-language journals. Studies were excluded if they did not provide sufficient qualitative data for synthesis or if they focused exclusively on animal models.

Data Extraction: Relevant information from each included study included author, year of publication, study design, population characteristics, and key qualitative findings related to neurodevelopmental or cognitive outcomes. Any discrepancies in data extraction were resolved through discussion or, when necessary, consultation with a third reviewer.

Qualitative Synthesis: Findings were mapped using a qualitative evidence synthesis approach. Thematic analysis was employed to identify and organize key patterns and themes across studies. A qualitative evidence map was generated to visually summarize the range of neurodevelopmental outcomes reported in CHD patients, highlighting areas of convergence and divergence within the literature.

## 3. Results

### 3.1. Single-Nucleus Analysis and Annotations Reveal Major Cardiac Cell Types in Congenital Heart Disease Patients

**Figure 1:**
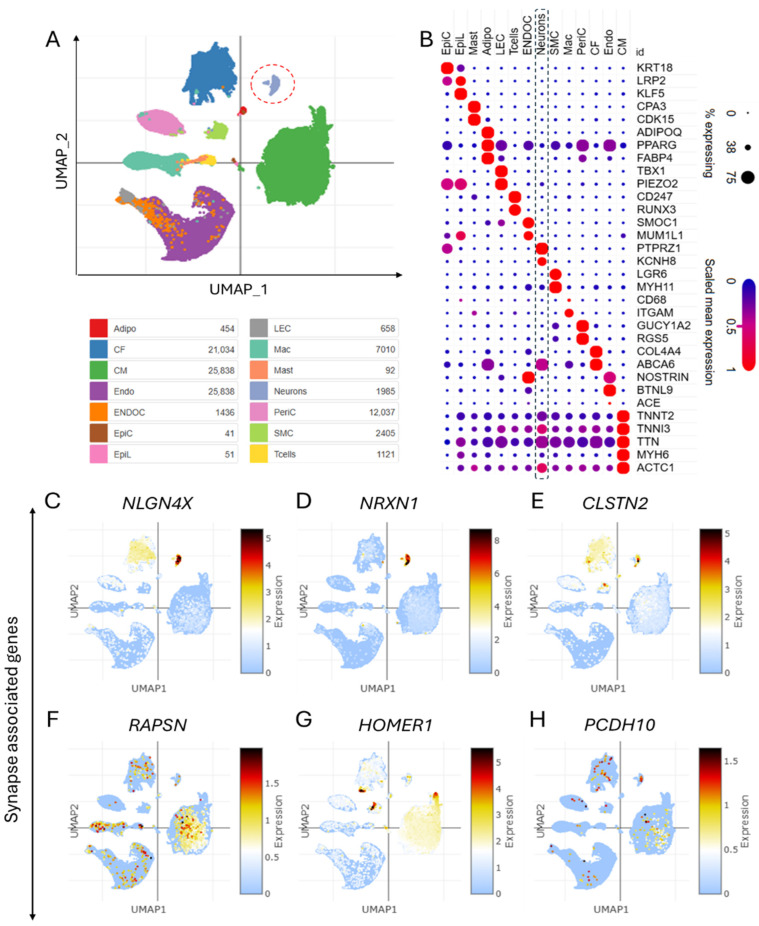
Single-cell transcriptomics identify diverse cardiac cell populations and neurocardiac synapses in congenital heart disease. (**A**) UMAP of the dataset showing different cell types in the heart tissues of congenital heart disease (CHD) patients. (**B**) Dot plot showing the cell type-specific markers-based annotation of different cell types in the heart tissues of CHD patients. (**C**–**H**) UMAPs show the cell type-specific expression of synapse-associated genes, including synaptic adhesion proteins encoding the genes *NLGN4X*, *NRXN1*, *CLSTN2*, *RAPSN*, *HOMER1*, and *PCDH10* in cardiac tissue of CHD.

Despite advancements in genomics, many CHD cases remain unexplained due to genetic heterogeneity and incomplete penetrance. Single-nucleus technologies address this gap by enabling high-resolution profiling of cellular heterogeneity and identifying molecular markers specific to cardiac cell subsets involved in CHD pathology [11,12,13]. Single-nucleus analysis and annotation techniques have proven instrumental in understanding the cellular landscape of CHD. In this study, single-nucleus transcriptomics analysis (GEO accession: GSE203274) [1] was employed to identify and characterize major cardiac cell types in CHD patients, offering new insights into the disease’s molecular and cellular basis. Figure 1A shows a Uniform Manifold Approximation and Projection (UMAP) visualization of heart tissue datasets from CHD patients, revealing distinct cardiac cell populations. Dot plot analysis enabled precise annotation of various cardiac cell types based on their unique gene expression profiles, providing a comprehensive map of the cellular landscape in CHD hearts (Figure 1B). Remarkably, we identified the expression of synaptic genes in cardiac tissues, suggesting the presence of neurocardiac connections in CHD patients. UMAPs revealed cell type-specific expressions of synaptic adhesion proteins and other synapse-associated genes (*NLGN4X*, *NRXN1*, *CLSTN2*, *RAPSN*, *HOMER1*, and *PCDH10*) in cardiac tissues (Figure 1C–H), indicating potential roles in heart development and function. The expression of these synaptic genes in cardiac cells suggests previously unrecognized communication pathways between the nervous system and the heart in CHD.

### 3.2. Gene Set Enrichment Analysis of Differentially Expressed Genes Enabled the Characterization of Cardiac Neurons in CHD

**Figure 2:**
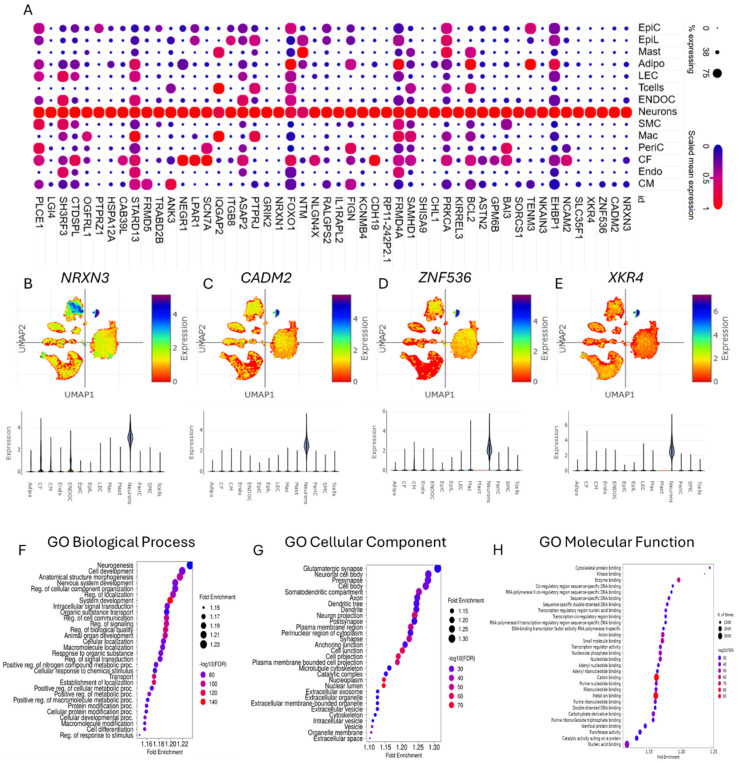
Characterization of cardiac neurons in congenital heart disease. (**A**) Dot plot depicting the expression of the top fifty genes predominantly found in neuron cell types in the heart. (**B**–**E**) UMAPs and violin plots showing the distribution of (**B**) *NRXN3*, (**C**) *CADM2*, (**D**) *ZNF536*, and (**E**) *XKR4* in different cell types with predominant expression in neuron cell types in the heart. (**F**–**H**) Enrichment plots showing the gene ontology (GO)-based major (**F**) biological processes, (**G**) cellular components, and (**H**) molecular functions of the neurons based on differentially expressed genes.

GSEA of differentially expressed genes could provide crucial insights into the characterization of cardiac neurons in CHD. The dot plot depicts the expression of the top 50 genes predominantly associated with neuronal cell types in the heart (Figure 2A), suggesting their specialized roles in cardiac neuronal function. Notably, UMAPs and violin plots reveal the distribution and predominant expression of key genes such as *NRXN3*, *CADM2*, *ZNF536*, and *XKR4* within cardiac neurons (Figure 2B–E), emphasizing their potential regulatory roles in CHD-related neuronal activity. Enrichment plots further elucidate gene ontology-based categorizations, identifying major biological processes, cellular components, and molecular functions linked to cardiac neurons (Figure 2F–H). Importantly, the neurogenesis and nervous system development are found to be the major biological processes associated with differentially expressed genes in cardiac neurons (Figure 2F). In addition, glutamatergic synapse was found to be one of the major cellular components associated with differentially expressed genes in cardiac neurons (Figure 2G). These findings accentuate the importance of understanding neuronal contributions to CHD pathology.

### 3.3. Genomic Landscape of CHD Features Upregulated Genes Linked to Cognitive Dysfunction and Neurodevelopment Disorder

**Figure 3:**
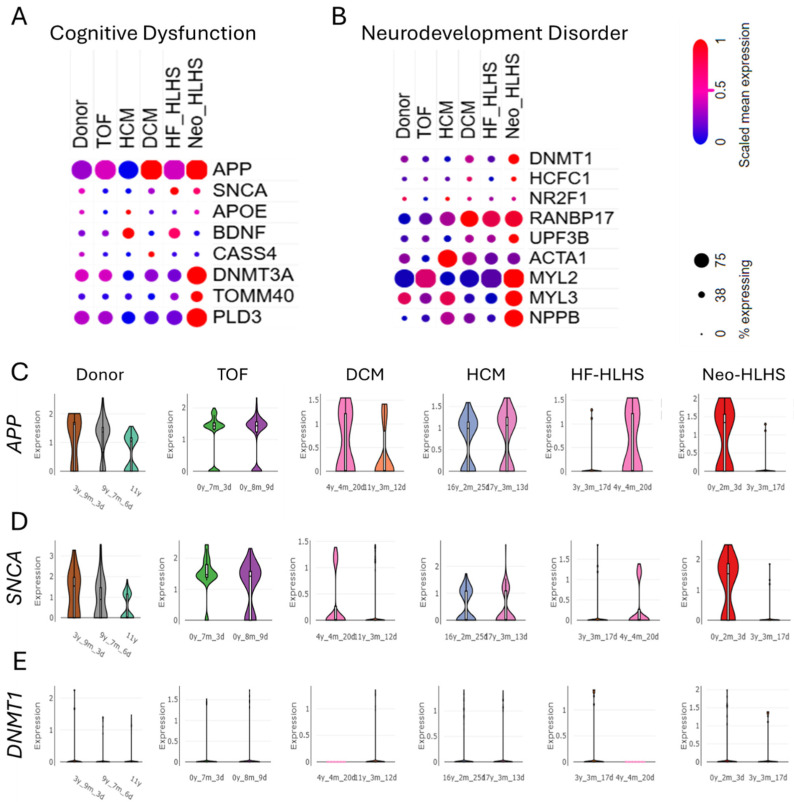
Genes linked to cognitive dysfunction are upregulated in congenital heart disease. (**A**,**B**) Dot plots showing the relative expression level of (**A**) cognitive dysfunction-associated genes *APP*, *SNCA*, *APOE*, *BDNF*, *CASS4*, *DNMTB3A*, *TOMM40*, and *PLD3* and (**B**) neurodevelopment disorder-associated genes *DNMT1*, *HCFC1*, *NR2F1*, *RANBP17*, *UPF3B*, *ACTA1*, *MYL2*, *MYL3*, *NPPB* in donor control, TOF, HCM, DCM, HF_HLHS, and Neo_HLHS. (**C**–**E**) Violin plots showing the age-dependent expression levels of *APP*, *SNCA*, *DNMT1* in control donor, TOF, HCM, DCM, HF_HLHS, and Neo_HLHS.

The genomic landscape of CHD reveals upregulated genes associated with cognitive dysfunction and neurodevelopmental disorders. Investigating these gene expression patterns is essential to uncover the mechanistic interplay between CHD and neurodevelopmental disorders, potentially guiding therapeutic strategies targeting both conditions. The dot plots illustrate the relative expression levels of cognitive dysfunction-associated genes (*APP, SNCA, APOE, BDNF, CASS4, DNMTB3A, TOMM40*, and *PLD3*) and neurodevelopmental disorder-associated genes (*DNMT1, HCFC1, NR2F1, RANBP17, UPF3B, ACTA1, MYL2, MYL3*, and *NPPB*) across various CHD subtypes, including TOF, HCM, DCM, HF_HLHS, and Neo_HLHS (Figure 3A,B). Supplementary Figure S1A–F depict the cell type-specific distribution of *APP, SNCA, APOE, BDNF, CASS4,* and *DNMTB3A* in donor control, TOF DCM, HCM, HF-HLHS, and Neo-HLHS. Further, violin plots further demonstrate age-dependent expression patterns of key genes (*APP, SNCA*, and *DNMT1*) in control donors and CHD subtypes (Figure 3C–E). These findings suggest a potential link between CHD pathology and neurological dysfunction, highlighting shared molecular pathways that may influence both cardiac and cognitive development.

### 3.4. Cardiac Troponin Elevation in CHD Signals Heart Attack Risk, Revealing Cell-Specific Crosstalk Between Cardiomyocytes and Neurons

**Figure 4:**
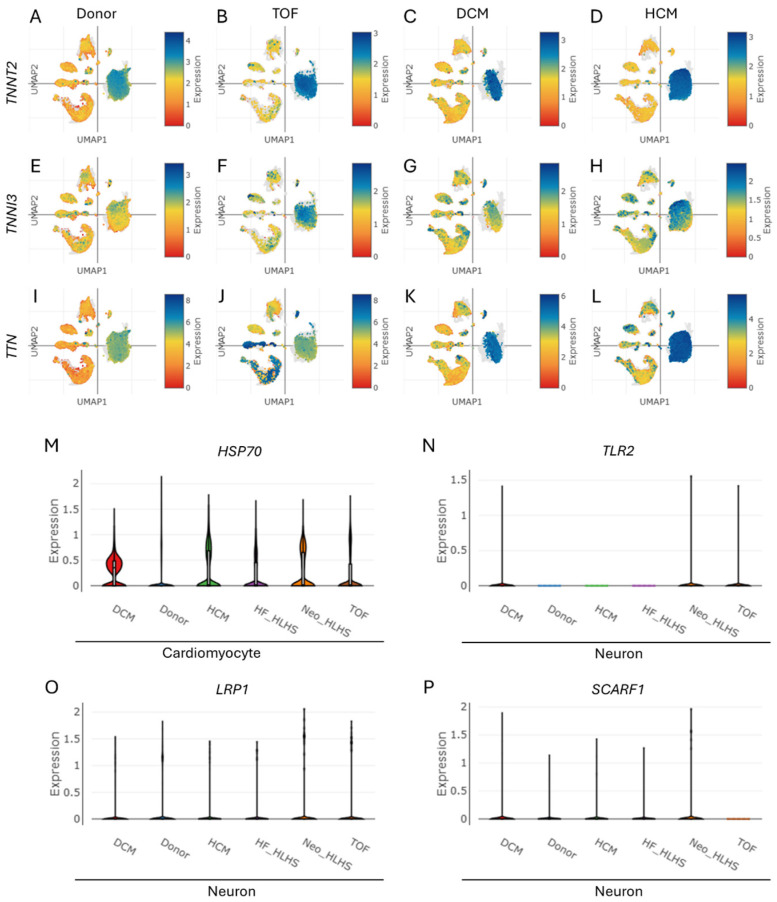
Cell type-specific expression level of troponin, predominantly enhanced in cardiomyocytes within CHD. (**A**–**L**) UMAPs show the enhanced expression levels of (**A**–**D**) TNNT2, (**E**–**H**) TNNI3, and (**I**–**L**) TTN in cardiomyocyte population within various CHDs, namely TOF, DCM, and HCM. (**M**) A violin plot depicting the enhanced expression level of HSP70 (associated with exosome biogenesis in the cardiomyocytes of TOF, HCM, DCM, HF_HLHS, and Neo_HLHS) compared to the donor control group. (**N**–**P**) Violin plots depicting the expression level of genes associated with exosome uptake receptors (**N**) TLR2, (**O**) LRP1, and (**P**) SCARF in the neuron cells of the donor control, TOF, HCM, DCM, HF_HLHS, and Neo_HLHS groups.

Cardiac troponin expression was analyzed across cardiomyocytes in CHD cohorts, including TOF, DCM, and HCM. UMAP visualizations demonstrated elevated expression of troponin genes *TNNT2* (Figure 4A–D), *TNNI3* (Figure 4E–H), and *TTN* (Figure 4I–L) in cardiomyocytes of TOF, DCM, HCM, HF-HLHS, and Neo-HLHS compared to the donor control (Supplementary Figure S2A–G). A violin plot revealed upregulated *HSP70* expression—a gene linked to exosome biogenesis—in cardiomyocytes of TOF, DCM, HCM, HF_HLHS, and Neo_HLHS versus donor controls (Figure 4M). Neuronal populations exhibited increased expression of exosome uptake receptors, with elevated *TLR2* (Figure 4N), *LRP1* (Figure 4O), and *SCARF* (Figure 4P) across all CHD subtypes. These findings suggest enhanced exosome-related signalling pathways in CHD, with cardiomyocytes showing increased troponin and exosome biogenesis markers, while neurons displayed upregulated receptors for exosome internalization. This suggests potential heart attack risk in CHD and exosome-based crosstalk between cardiomyocytes and neurons.

### 3.5. Troponin Levels in CHD Correlate with Markers of Cognitive Dysfunction and Neurodevelopment Disorder

**Figure 5:**
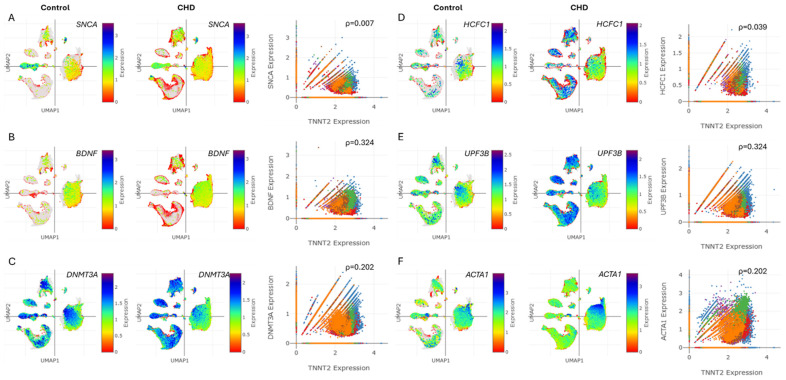
Cognitive dysfunction and neurodevelopment markers are associated with troponin levels in CHD. (**A**–**F**) UMAPs depicting the cell type-specific expression of *SNCA, BDNF, DNMT3A, HCFC1, UPF3B,* and *ACTA1* in the heart for the control and CHD groups. (**A**–**F**) Correlation plots showing the correlation for (**A**) *SNCA* vs. *TNNT2*, (**B**) *BDNF* vs. *TNNT2*, (**C**) *DNMT3A* vs. *TNNT2*, (**D**) *HCFC1* vs. *TNNT2*, (**E**) *UPF3B* vs. *TNNT2*, and (**F**) *ACTA1* vs. *TNNT2* in cells from the hearts of the control and CHD patients. ρ depicts the quantitative correlation coefficient.

CHD patients face higher risks of cognitive, motor, language, and social–emotional challenges. These neurodevelopmental issues can affect executive functions and overall development [8]. Therefore, we aimed to investigate the relationship between *TNNT2* and markers of cognitive dysfunction and neurodevelopment disorder (NDD) in both control and CHD heart cells. The UMAPs show the cell type-specific distribution and expression levels of *SNCA*, *BDNF*, *DNMT3A*, *HCFC1*, *UPF3B*, and *ACTA1* in the heart for the control and CHD groups. The snRNA-seq analysis revealed significant correlations between *TNNT2* expression in CHD and markers linked to cognitive dysfunction and neurodevelopment (Figure 5A–F, Supplementary Figure S3A–D). Positive associations were observed between *TNNT2* and *SNCA* (ρ = 0.007), *BDNF* (ρ = 0.324), DNMT3A (ρ = 0.202), *HCFC1* (ρ = 0.039), *UPF3B* (ρ = 0.324), and *ACTA1* (ρ = 0.202) (Figure 5A–F). These findings align with clinical studies demonstrating troponin’s association with cognitive decline and neurodevelopmental disorder [14,15].

### 3.6. Ontology of Differentially Expressed Genes in Cardiac Neurons of CHD Suggests Links Between Cardiovascular Disease Phenotypes and Neurodevelopmental Disorders

**Figure 6:**
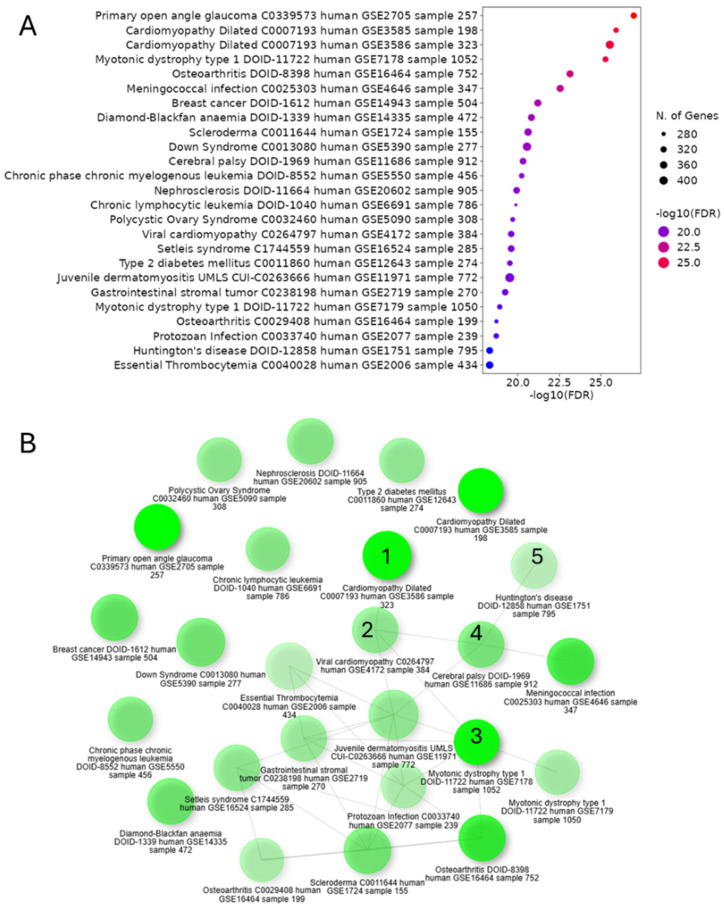
Cardiac neuronal gene expression in CHD indicates connections between cardiovascular phenotypes and neurodevelopmental disorders. (**A**) GSEA-based dot plot showing the various disease conditions, as identified through the enrichment of differentially upregulated genes in cardiac neurons of CHD, indicating (**B**) the association between cardiovascular disease phenotypes (marked as 1 and 2) and neurodevelopment disorders (marked as 3–5), as shown in the GSEA-based network graph.

We aimed to investigate potential connections between CHD and NDD by analyzing gene expression patterns in cardiac neurons from CHD patients, addressing the increased risk of neurodevelopmental issues in CHD survivors. GSEA of differentially upregulated genes in cardiac neurons from CHD patients revealed significant associations with various cardiovascular phenotypes (including ventricular septal defects, atrial septal defects, Tetralogy of Fallot, and cardiomyopathies) and neurodevelopmental disorders (such as autism spectrum disorders, intellectual disability, developmental delay, and epilepsy) (Figure 6A). The GSEA-based network graph illustrates the complex interplay between cardiovascular phenotypes and neurodevelopmental disorders, with key nodes representing the shared genetic pathways and molecular mechanisms underlying both CHD and NDD (Figure 6B). These findings suggest a potential common genetic etiology between CHD and NDD, with cardiac neuronal gene expression patterns reflecting both cardiovascular and neurodevelopmental abnormalities.

### 3.7. System Meta-Analysis Revealed Clinical Association Between CHD and Neurodevelopmental Disorders

**Figure 7:**
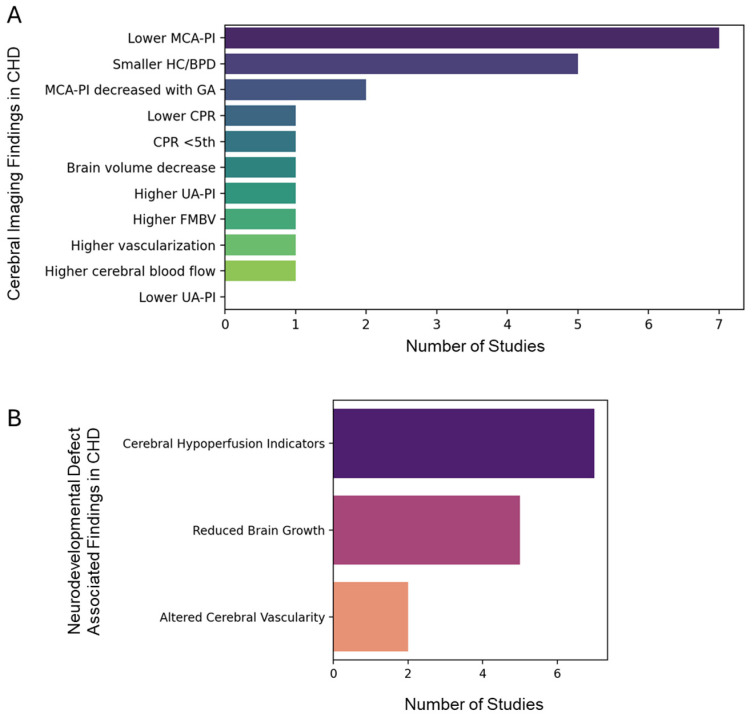
Qualitative evidence map of clinical findings in congenital heart disease (CHD). Bar graphs illustrate the number of studies reporting (**A**) prenatal ultrasound findings and (**B**) imaging findings associated with neurodevelopmental impairment in CHD. Note: MCA-PI: middle cerebral artery pulsatility index; CPR: cerebro-placental ratio; HC/BPD: head circumference or biparietal diameter; UA-PI: umbilical artery PI; FMBV: fractional moving blood volume.

To address the need for a comprehensive understanding of the neurodevelopmental risks associated with CHD, as previous individual studies reported varying results (Supplementary Table S1) [16,17,18,19,20,21,22,23,24,25,26,27,28,29,30,31,32,33,34,35,36], we performed a systematic analysis. Interestingly, the qualitative evidence map presented in Figure 7A,B synthesizes clinical findings related to CHD, focusing on prenatal ultrasound findings and imaging findings associated with neurodevelopmental impairment. The bar graphs quantitatively display the frequency with which specific clinical features are reported across the published studies, offering a visual summary of the evidence landscape, based on a previous studies (Supplementary Tables S1 and S2). Notably, certain prenatal ultrasound markers are more frequently identified in the literature, underscoring their perceived clinical relevance for early CHD detection and risk stratification [37]. The map also highlights imaging findings—such as structural brain abnormalities or altered brain growth patterns—that are recurrently associated with neurodevelopmental impairment in children with CHD [38]. Further, the expression analysis of genes associated with familial Alzheimer’s disease (AD), sporadic AD, and Parkinson’s disease (PD) were found to be relatively upregulated in CHD as compared to the control donor (Supplementary Figure S4), suggesting the common link between the neurodegenerative and CHD genomic landscape. Also, the profile of serum- or cell-free DNA biomarkers of CHD (Supplementary Figure S5) showed the possibility of utilizing biofluids, including serum or amniotic fluid, for potential CHD liquid biopsy-based diagnosis.

## 4. Discussion

Single-nucleus transcriptomic analyses have significantly advanced our understanding of cellular heterogeneity in CHD, addressing gaps left by bulk sequencing. Studies using snRNA-seq have identified distinct cardiac cell populations, including dysregulated progenitor subtypes and chamber-specific transcriptional signatures, which correlate with CHD pathology [12,39,40,41]. For instance, Hand2-null models revealed failed outflow tract specification and disrupted retinoic acid signalling in progenitors, while chamber-specific cell cycle dynamics were linked to growth defects [42]. The UMAP visualization and marker-based annotation in the described study (Figure 1A,B) align with established workflows for resolving cardiomyocyte-, endothelium-, and neural crest-derived subsets, as seen in human fetal heart atlases [12,40]. Moreover, the expression of synaptic genes in cardiac tissues of CHD patients (Figure 1C–H) suggests previously unrecognized communication pathways between the nervous system and the heart.

Single-nucleus analyses have revealed significant overlap between cardiac neuronal gene expression and CHD pathology, with GSEA highlighting neurogenesis, nervous system development, and glutamatergic synapse pathways as central to cardiac neuron dysfunction. Genes like *NRXN3*, *CADM2*, and *HCN1* (implicated in synaptic adhesion and ion channel activity) show enriched expression in CHD-associated cardiac neurons (Figure 2A–E), aligning with prior reports linking neural crest cell migration defects to outflow tract malformations [43,44,45]. These findings mirror pathway enrichments observed in CNV studies of CHD cohorts, where neuronal system genes (*ADCY2*, *NRXN3*) correlate with conotruncal defects [43,46]. The glutamatergic synapse signature (Figure 2G) parallels recent snRNA-seq data showing neurotransmitter signalling dysregulation in CHD cardiomyocytes [47].

Recent genomic analyses of CHD have revealed a complex interplay between cardiac malformations and NDDs. The upregulation of genes associated with cognitive dysfunction and NDDs in CHD patients aligns with previous findings linking de novo mutations in heart-expressing genes to both CHD and NDD phenotypes [10]. This genetic overlap supports the “burden of genetic variation” model, where multiple rare variants contribute to both cardiac and neurological outcomes [48]. The expression patterns of genes like *APP*, *SNCA*, and *DNMT1* across CHD subtypes and age groups (Figure 3A–E) suggest shared molecular pathways influencing cardiac and cognitive development. This corroborates recent studies identifying 60 genes implicated in CHD, with many also contributing to autism and other NDDs [46]. The involvement of chromatin modifiers in both CHD and NDD further emphasizes the genetic link between heart and brain development [49,50].

We found the elevated cardiac troponin expression (*TNNT2*, *TNNI3*, *TTN*) in cardiomyocytes across various CHD subtypes, suggesting increased myocardial stress and potential heart attack risk (Figure 4A–L). This aligns with a previous clinical study showing higher troponin levels in CHD patients, indicating myocardial injury [51]. The upregulation of *HSP70* in cardiomyocytes (Figure 4M) further supports this, as *HSP70* is associated with cellular stress responses and exosome biogenesis [52]. Although the precise mechanism underlying elevated troponin gene expression in CHD is not fully understood, one possible explanation is that the accumulation of amyloid fibrils—potentially originating from APP—induces myocardial injury, which in turn contributes to troponin elevation [53].

Interestingly, neuronal populations in CHD subtypes exhibited increased expression of exosome uptake receptors (*TLR2*, *LRP1*, *SCARF*) (Figure 4N–P), suggesting enhanced exosome-mediated crosstalk between cardiomyocytes and neurons. This novel finding extends our understanding of cell–cell communication in CHD beyond the established endothelial cell–cardiomyocyte interactions [52,54]. Further, significant correlations were found between *TNNT2* expression and markers of cognitive dysfunction (Figure 5A–C, Supplementary Figure S3A,B) and NDDs (Figure 5D–F, Supplementary Figure S3C,D) in CHD patients. This aligns with recent clinical reports showing associations between elevated cardiac troponin levels and cognitive decline, dementia risk, and neurodevelopmental issues in CHD patients [55,56]. The positive correlations between *TNNT2* and genes like *SNCA*, *BDNF*, and *DNMT3A* (Figure 5A–C) suggest shared pathways between cardiac dysfunction and neurological processes. This supports the “brain–heart axis” concept, where cardiac biomarkers may serve as early indicators of cognitive vulnerability in CHD [55]. The association with *HCFC1* and *UPF3B* (Figure 5D,E) further emphasizes the link between CHD and NDDs, consistent with previous findings of overlapping genetic factors [57,58].

Interestingly, the GSEA of differentially upregulated genes in cardiac neurons from CHD patients reveals significant associations between cardiovascular phenotypes and NDDs (Figure 6A). This finding aligns with previous studies demonstrating shared genetic contributions to CHD and NDD. For instance, exome sequencing of CHD parent–offspring trios identified an excess of protein-damaging de novo mutations in genes highly expressed in both the developing heart and brain [59]. These mutations accounted for 20% of patients with CHD, NDD, and extra-cardiac congenital anomalies, suggesting pleiotropic effects. Further, the complex interplay between cardiovascular phenotypes and NDDs, illustrated in the GSEA-based network graph (Figure 6A,B), supports the “brain–heart axis” concept. This is consistent with recent findings showing that CHD patients have an increased risk of neurodevelopmental disabilities, with up to 33% of CHD patients with CNVs also having an associated NDD [60]. Our analysis identifies nine major cardiac cell populations, with neuronal clusters showing striking enrichment for NDD-associated genes (*NRXN3*, *CADM2*, *ZNF536*) and synaptic signalling pathways. These neurons co-express cognitive dysfunction markers (*APP*, *SNCA*) and chromatin modifiers (*DNMT1*, *HCFC1*) across CHD subtypes, suggesting maintained neurodevelopmental potential postnatally. Parallel findings reveal cardiomyocyte-specific troponin upregulation correlated with neuronal exosome receptor expression (*TLR2*, *LRP1*), supported by strong *SNCA–TNNT2* correlations. Gene ontology analysis demonstrates significant overlap between cardiovascular disease pathways and neurodevelopmental disorder signatures, particularly in Wnt/β-catenin signalling and histone modification modules. In addition, the systemic review and meta-analysis further demonstrated the importance of integrating neurodevelopmental surveillance into routine follow-up for these patients (Figure 7A,B). The evidence map identifies both well-established and underexplored clinical markers, guiding clinicians and researchers toward areas where evidence is robust and highlighting gaps where further investigation is warranted. This approach supports more targeted allocation of resources for diagnosis, intervention, and long-term neurodevelopmental monitoring in CHD populations [37,38]. In conclusion, this study reveals significant links between CHD and NDD through single-nucleus transcriptomics, highlighting shared genetic pathways and potential biomarkers. These findings emphasize the importance of integrated cardiac and neurological care for CHD patients, paving the way for targeted interventions and improved outcomes.

Despite this, this study faces several limitations that impact the interpretation and generalizability of its findings. These include challenges in integrating data across different platforms and limited spatial resolution, which hinder a comprehensive understanding of cell–cell interactions. The reliance on transcriptomic data without functional or electrophysiological validation leaves gaps in understanding the causal relationships between gene expression patterns and the observed phenotypes. The study’s cross-sectional nature precludes establishing causality, while potential confounding factors such as age, medication, comorbidities, hypoxia, and surgical interventions are not fully accounted for. Additionally, the incomplete capture of non-coding variants, epigenetic factors, and somatic mosaicism may overlook important regulatory mechanisms. The analysis also lacks sufficient resolution to fully elucidate spatial interactions between cardiac neurons and other cell types. These limitations pave the way for future studies elucidating various aspects of neurodevelopmental defects in CHD.

## 5. Conclusions

CHD patients face significant neurodevelopmental risks, including cognitive delays, motor impairments, and motor dysfunction, which persist across the lifespan. Research indicates lower cognitive abilities, particularly in severe CHD subtypes like HLHS, alongside vulnerabilities in attention, memory, and adaptive functioning. These deficits stem from altered cerebral blood flow, genetic factors, and brain immaturity, with white matter injury and stroke further exacerbating outcomes. Emerging evidence highlights shared molecular pathways between CHD and neurodevelopmental disorders, suggesting cardiac–neuronal crosstalk via exosomes. Early monitoring and multidisciplinary interventions are critical to mitigating long-term impacts on education, employment, and independent living.

## Data Availability

The data presented in this study are available in a GEO public database at https://www.ncbi.nlm.nih.gov/geo/ (accessed on 24 January 2025), reference number [GEO accession: GSE203274]. These data were derived from the following resources available in the public domain: https://doi.org/10.1038/s41586-022-04989-3 [1].

## Supplementary Materials

The following supporting information can be downloaded at https://www.mdpi.com/article/10.3390/life15091400/s1: Figure S1: Identification of cognitive markers’ cell type-specific expression level in control and CHD hearts; Figure S2: Evaluation of cardiac troponin in CHD; Figure S3: Correlation analysis of markers of cognitive dysfunction and neurodevelopment markers with troponin levels in CHD. Figure S4: Genes linked to AD and PD are upregulated in congenital heart disease. Figure S5: Expression analysis of genes encoding serum- or cell-free DNA markers of CHD. Table S1: A list of studies showing key prenatal and preoperative cerebral imaging findings in CHD. Table S2: A list of studies showing neurodevelopment defect-relevant ultrastructure markers in CHD.

## Abbreviations

The following abbreviations are used in this manuscript:

CHD	Congenital heart disease
HLHS	Hypoplastic left heart syndrome
TOF	Tetralogy of Fallot
DCM	Dilated cardiomyopathy
HCM	Hypertrophic cardiomyopathy
UMAP	Uniform Manifold Approximation and Projection
NRXN3	Neurexin 3
CADM2	Cell adhesion molecule 2
ZNF536	Zinc finger protein 536
APP	Amyloid precursor protein
SNCA	Synuclein alpha
BDNF	Brain-derived neurotrophic factor
DNMT1	DNA methyltransferase 1
HCFC1	Host cell factor C1
TLR2	Toll-like receptor 2
LRP1	Low-density lipoprotein receptor-related protein 1
NDDs	Neurodevelopmental disorders
DNMT3A	DNA methyltransferase 3 alpha
GEO	Gene Expression Omnibus
NF-κB	Nuclear factor kappa-light-chain-enhancer of activated B cells
