# Supplementary material for: Neuro-Genomic Mapping of Cardiac Neurons with Systemic Analysis Reveals Cognitive and Neurodevelopmental Impacts in Congenital Heart Disease
Source: Life (Basel). 2025 Sep 4;15(9):1400. doi: 10.3390/life15091400 (PMC12471760; doi:10.3390/life15091400)
Supplement: Supplementary file 1 [file life-15-01400-s001.zip › life-3829615-supplementary.pdf]

Supplementary Data

Neuro-genomic Mapping of Cardiac Neurons with Systemic Meta-analysis Reveals Cognitive and Neurodevelopmental Impacts in Congenital Heart Disease

Abhimanyu Thakur[1], Raj Kishore[1,2*]

[1]Aging and Cardiovascular Discovery Center, Lewis Katz School of Medicine, Temple University, Philadelphia, PA 19140, USA.
[2]Department of Cardiovascular Sciences, Lewis Katz School of Medicine, Temple University, Philadelphia, PA 19140, USA.

[*]Corresponding author: raj.kishore@temple.edu (R.K.)

Supplementary Figures

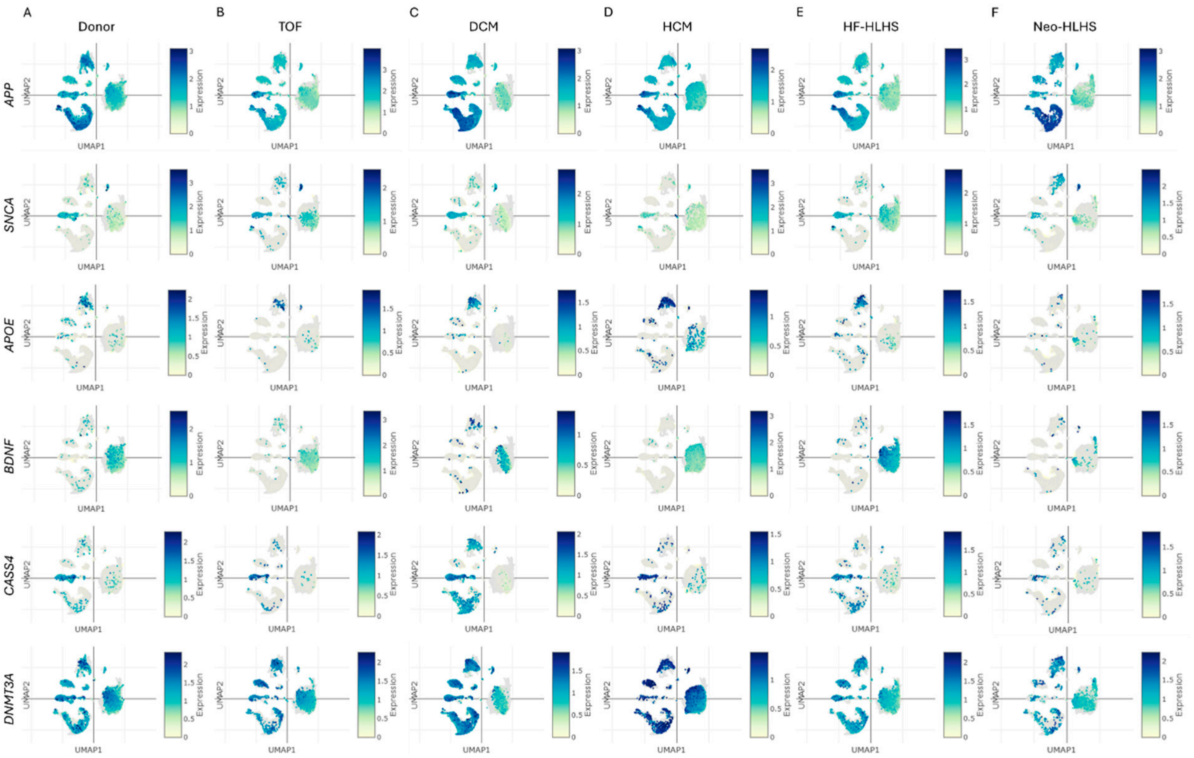

Supplementary Fig. S1. Identification of cognitive markers' cell type-specific expression level in control and CHD hearts. UMAPs depicting cell type-specific distribution of *APP*, *SNCA*, *APOE*, *BDNF*, *CASS4*, and *DNMTB3A* in **(A)** donor control, **(B)**TOF, **(C)** DCM, **(D)** HCM, **(E)** HF-HLHS, and **(F)** Neo-HLHS.

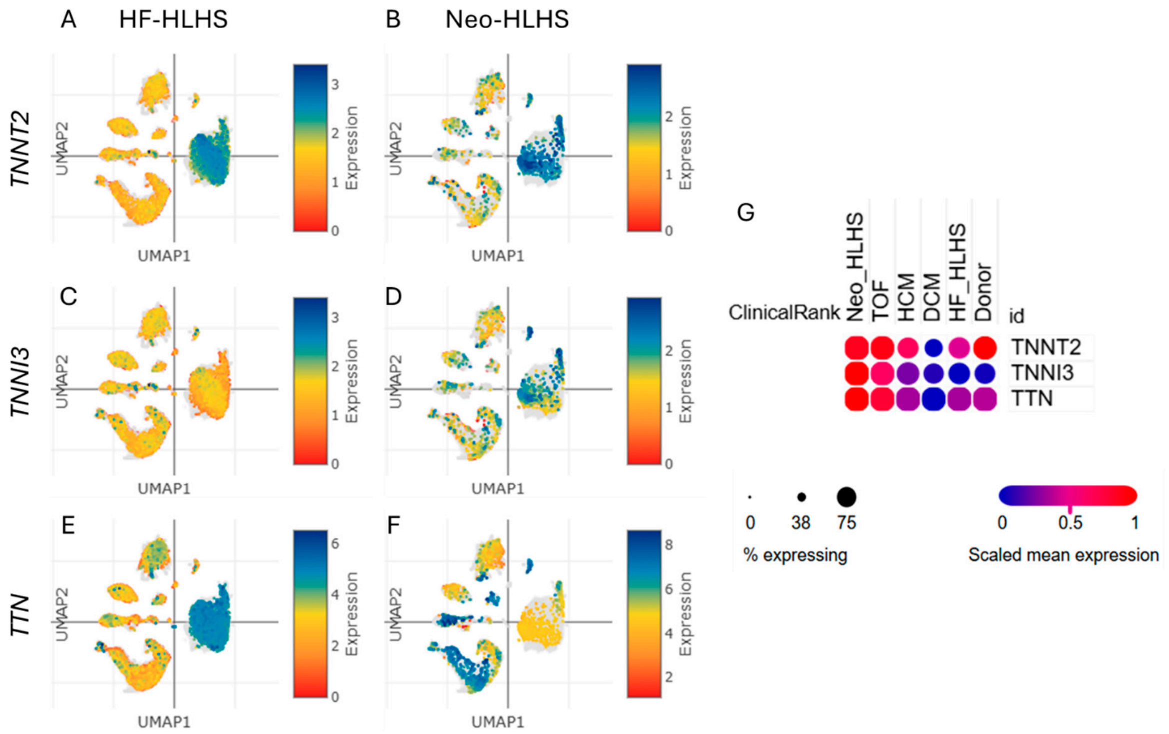

Supplementary Fig. S2. Evaluation of cardiac troponin in CHD. (A-F) UMAPs show the cell type-specific expression of (A-B) *TNNT2*, (C-D) *TNNI3*, and (E-F) *TTN* in the heart of HF-HLHS and Neo-HLHS. **(G)** Dot plot shows the expression level of *TNNT2*, *TNNI3*, and *TTN* in donor control, TOF, DCM, HCM, HF-HLHS and Neo-HLHS.

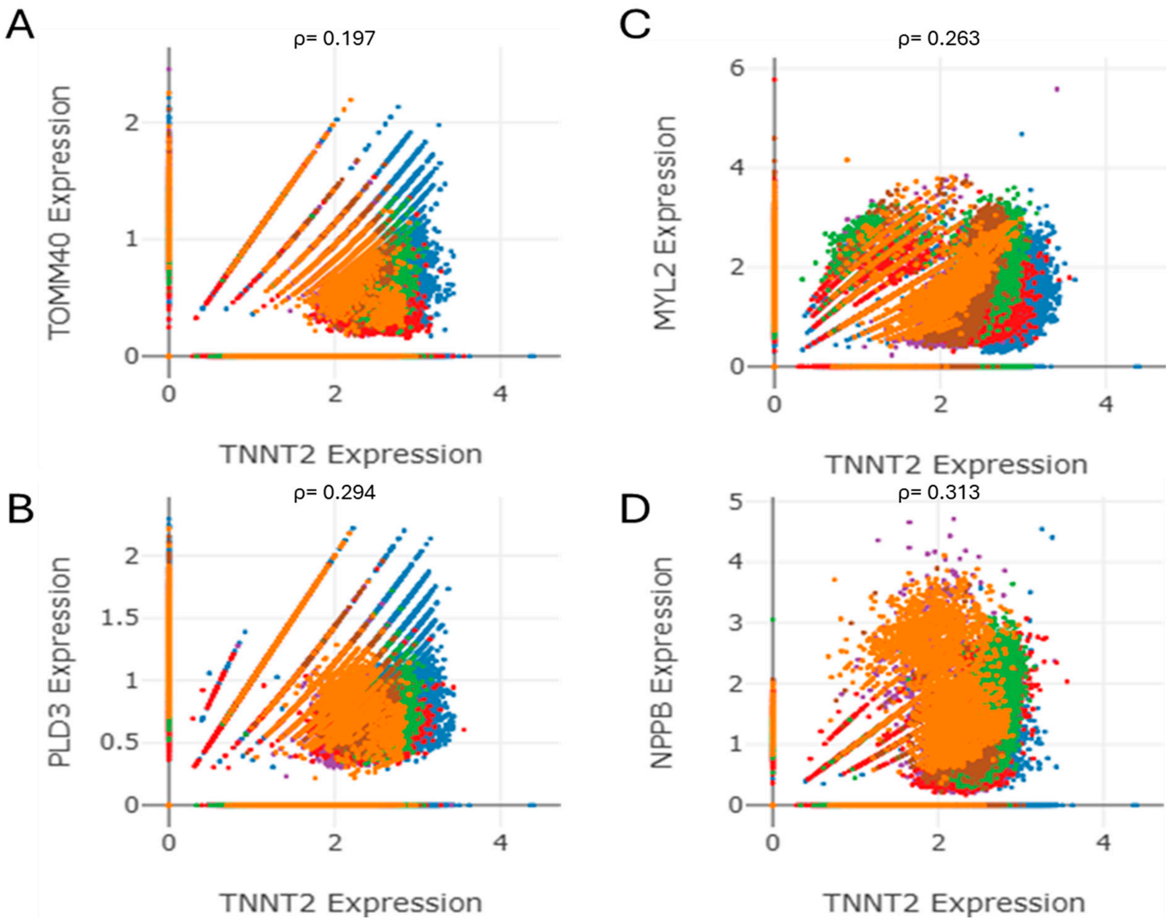

Supplementary Fig. S3. Correlation analysis of markers of cognitive dysfunction and neurodevelopment markers with troponin levels in CHD. Correlation plots showing the correlation for (A) *TOMM40* vs. *TNNT2,* (B) *PLD3* vs. *TNNT2,* (C) *MYL2* vs. *TNNT2*, and (D) *NPPB* vs. *TNNT2* in cells from the heart of control and CHD patients. ϱ depicts the quantitative correlation coefficient.

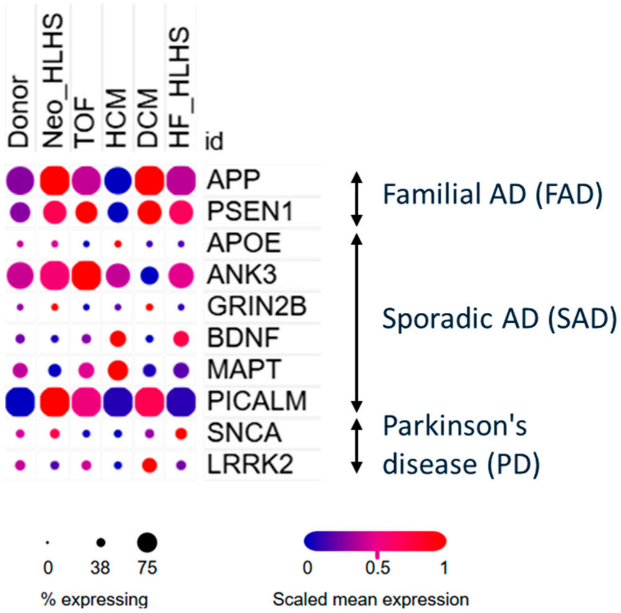

Supplementary Fig. S4. Genes linked to AD and PD are upregulated in congenital heart disease. Dot plots showing the relative expression level of familial AD (*APP, and PSEN1*), sporadic AD (*APOE, ANK3, GRIN2B, BDNF, MAPT, and PICALM*), and Parkinson's disease (*SNCA and LRRK2*).

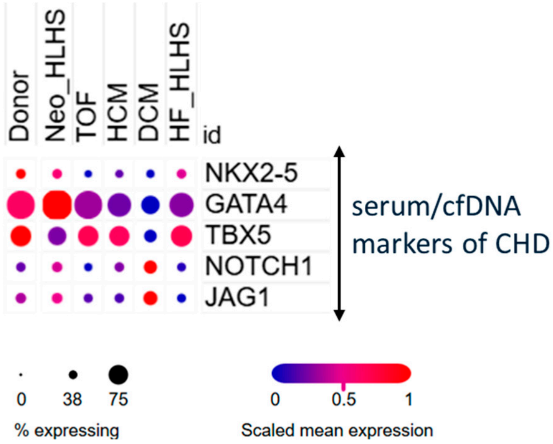

Supplementary Fig. S5. Expression analysis of genes encoding serum- or cell-free DNA markers of CHD. Dot plots show the relative expression level of genes associated with serum- or cell-free DNA markers of CHD.

Supplementary Table S1. A list of studies showing key prenatal and preoperative cerebral imaging findings in CHD.

Study	Study Design, Sample Size	CHD Type	Timing/Age	Methods	Main Findings (vs. Controls/Reference)	Ref.
Ultrasound Findings						
Ruiz et al, Ultrasound Obstet Gynecol, 2017	Retrospective, N=119	Mixed	2nd/3rd trimester	Ultrasound (biometry, Doppler)	18% had MCA-PI and CPR <5th percentile at first exam; lower MCA-PI in severe cerebral blood flow impairment; smaller HC/BPD at diagnosis persisted throughout pregnancy	[1]
Hahn et al, Ultrasound Obstet Gynecol, 2016	Retrospective, N=133	SVA	2nd/3rd trimester	Ultrasound (biometry, Doppler)	Lower MCA-PI, decreased with GA; smaller HC at 24–29 and >34 wk; fetal HC predicted neonatal HC; MCA-PI not linked to HC	[2]
Zeng et al, Ultrasound Obstet Gynecol, 2015	Case–control, N=73/168	Mixed	2nd/3rd trimester	Ultrasound (biometry, Doppler)	Lower MCA-PI; progressive decrease in brain volumes from 28 wk; largest in frontal lobe; smaller HC/BPD from 33 wk	[3]
Zeng et al, Ultrasound Obstet Gynecol, 2015	Case–control, N=112/112	Mixed	20–30 wk	Ultrasound (Doppler)	Lower MCA-PI in HLHS; higher cerebral blood flow in HLHS/LSOL; higher vascularization indices in HLHS/LSOL/TGA	[4]
Masoller et al, Ultrasound Obstet Gynecol, 2014	Case–control, N=95/95	Mixed	20–24 wk	Ultrasound (biometry, Doppler)	Lower MCA-PI/CPR; higher fractional moving blood volume (>95th percentile in 81% vs. 11% controls); smaller BPD/HC	[5]
Williams et al, Am Heart J, 2013	Cohort, N=134	SVA	18–38 wk	Ultrasound (Doppler)	MCA-PI at first echo −0.95±1.5; 22% had MCA-PI <−2.0 at least once	[6]
Yamamoto et al, Ultrasound Obstet Gynecol, 2013	Case–control, N=89/89	Mixed	32 wk	Ultrasound (biometry, Doppler)	Lower MCA-PI, higher UA-PI, lower CPR in HLHS/CoA; smaller HC at birth in TGA/CoA	[7]

Study	Study Design, Sample Size	CHD Type	Timing/Age	Methods	Main Findings (vs. Controls/Reference)	Ref.
Szwast et al., Ultrasound Obstet Gynecol, 2012	Retrospective, N=131/92	SVA	18–40 wk	Ultrasound (Doppler)	Lower MCA-PI/CPR in aortic arch obstruction vs. controls/pulmonary obstruction; MCA-PI decreased with gestation in aortic obstruction	[8]
Williams et al., Ultrasound Obstet Gynecol, 2012	Pilot, N=13	Mixed	20–24 wk	Ultrasound (Doppler)	MCA-PI −1.7±1.1; 56% CPR <1.0; HLHS/TOF lowest MCA-PI	[9]
Arduini et al., J Matern Fetal Neonatal Med, 2011	Case–control, N=60/65	Mixed	30–38 wk	Ultrasound (biometry, Doppler)	Lower MCA-PI/CPR; HLHS/CoA lowest, TOF/TGA highest CPR; smaller HC/HC-AC	[10]
McElhinney et al., Ultrasound Med Biol, 2010	Cohort, N=52	HLHS	20–31 wk	Ultrasound (Doppler)	Lower MCA-PI/RI in HLHS; 37% CPR <1.0	[11]
Berg et al., Ultrasound Obstet Gynecol, 2009	Case–control, N=113/1378	Mixed	19–41 wk	Ultrasound (biometry, Doppler)	Smaller HC at birth, normal MCA-PI/CPR in TGA; smaller HC, lower MCA-PI/CPR in HLHS	[12]
MRI Findings						
Brossard-Racine et al., Am J Neuroradiol, 2016	Cohort, N=103	Mixed	2nd/3rd trimester	MRI (structural)	16% fetal brain abnormalities; 32% neonatal brain abnormalities (27% acquired); mainly punctate white matter injury	[13]
Brossard-Racine et al., Am J Neuroradiol, 2014	Case–control, N=144/194	Mixed	18–39 wk	MRI (structural)	23% brain injury in CHD vs. 1.5% controls; most common: mild unilateral ventriculomegaly, increased extra-axial CSF	[14]
Mlczoch et al., Eur J Paediatr Neurol, 2013	Retrospective, N=53	Mixed	20–37 wk	MRI (structural)	39% brain injury (malformation, acquired lesion, asymmetry)	[15]
Schellen et al., Am J Obstet Gynecol, 2015	Retrospective, N=24/24	TOF	25 wk	MRI (volume)	Lower total/cortical/subcortical brain volume; higher ventricular/CSF spaces	[16]

Study	Study Design, Sample Size	CHD Type	Timing/Age	Methods	Main Findings (vs. Controls/Reference)	Ref.
Al Nafisi et al., J Cardiovasc Magn Reson, 2013	Case–control, N=22/12	Mixed	30–39 wk	MRI (volume)	6 fetus brain weights <5th percentile; 19% lower combined ventricular output	[17]
Sun et al., Circulation, 2015	Case–control, N=30/30	Mixed	36 wk	MRI (volume, O2)	Smaller brain volume; 15% ↓ cerebral O2 delivery, 32% ↓ O2 consumption; 13% ↓ brain volume	[18]
Limperopoulos et al., Circulation, 2010	Case–control, N=55/50	Mixed	25–37 wk	MRI (volume, metabolism)	Smaller total brain/intracranial volumes; lower NAA/Cho; cerebral lactate in 7 CHD fetuses	[19]
Masoller et al., Fetal Diagn Ther, 2016	Case–control, N=58/58	Mixed	36–38 wk	US (Doppler), MRI (volume, metabolism)	Lower MCA-PI/CPR, higher frontal moving blood volume; smaller brain/intracranial volume, decreased sulcation, altered metabolism	[20]
Clouchoux et al., Cereb Cortex, 2013	Case–control, N=18/30	HLHS	25–37 wk	US (Doppler), MRI (volume)	Smaller brain volumes, sulcation delay; low CPR, absence of antegrade flow linked to decreased cortical/subcortical matter	[21]

Abbreviations: MCA-PI: middle cerebral artery pulsatility index; CPR: cerebroplacental ratio; HC: head circumference; BPD: biparietal diameter; SVA: single-ventricle anomaly; HLHS: hypoplastic left heart syndrome; TGA: transposition of the great arteries; TOF: Tetralogy of Fallot; LSOL/RSOL: left-/right-sided outflow lesion; UA-PI: umbilical artery pulsatility index; WMI: white matter injury; PVL: periventricular leukomalacia; CSF: cerebrospinal fluid; NAA/Cho: N-acetylaspartate/choline ratio.

Supplementary Table S2. A list of studies showing neurodevelopment defect-relevant ultrastructure markers in CHD.

Study	Cerebral Hypoperfusion Indicators	Reduced Brain Growth	Altered Cerebral Vascularity
Ruiz et al 2017	1	1	0
Hahn et al 2016	1	1	0
Zeng et al 2015a	1	1	0
Zeng et al 2015b	1	0	1
Masoller et al 2014	1	1	1
Williams et al 2013	0	0	0

Yamamoto et al 2013	1	1	0
Szwast et al 2012	1	0	0

References

1. Ruiz A, Cruz-Lemini M, Masoller N, Sanz-Cortés M, Ferrer Q, Ribera I, et al. Longitudinal changes in fetal biometry and cerebroplacental hemodynamics in fetuses with congenital heart disease. Ultrasound Obstet Gynecol [Internet]. 2017;49:379–86. Available from: https://obgyn.onlinelibrary.wiley.com/doi/10.1002/uog.15970
2. Hahn E, Szwast A, Cnota J, Levine JC, Fifer CG, Jaeggi E, et al. Association between fetal growth, cerebral blood flow and neurodevelopmental outcome in univentricular fetuses. Ultrasound Obstet Gynecol [Internet]. 2016;47:460–5. Available from: https://obgyn.onlinelibrary.wiley.com/doi/10.1002/uog.14881
3. Zeng S, Zhou QC, Zhou JW, Li M, Long C, Peng QH. Volume of intracranial structures on three-dimensional ultrasound in fetuses with congenital heart disease. Ultrasound Obstet Gynecol [Internet]. 2015;46:174–81. Available from: https://obgyn.onlinelibrary.wiley.com/doi/10.1002/uog.14677
4. Zeng S, Zhou J, Peng Q, Tian L, Xu G, Zhao Y, et al. Assessment by three-dimensional power Doppler ultrasound of cerebral blood flow perfusion in fetuses with congenital heart disease. Ultrasound Obstet Gynecol [Internet]. 2015;45:649–56. Available from: https://onlinelibrary.wiley.com/doi/10.1002/uog.14798
5. Masoller N, Martínez JM, Gómez O, Bennasar M, Crispi F, Sanz-Cortés M, et al. Evidence of second-trimester changes in head biometry and brain perfusion in fetuses with congenital heart disease. Ultrasound Obstet Gynecol [Internet]. 2014;44:182–7. Available from: https://obgyn.onlinelibrary.wiley.com/doi/10.1002/uog.13373
6. Williams IA, Fifer C, Jaeggi E, Levine JC, Michelfelder EC, Szwast AL. The association of fetal cerebrovascular resistance with early neurodevelopment in single ventricle congenital heart disease. Am Heart J [Internet]. 2013;165:544-550.e1. Available from: https://linkinghub.elsevier.com/retrieve/pii/S0002870313000562
7. Yamamoto Y, Khoo NS, Brooks PA, Savard W, Hirose A, Hornberger LK. Severe left heart obstruction with retrograde arch flow influences fetal cerebral and placental blood flow. Ultrasound Obstet Gynecol [Internet]. 2013;42:294–9. Available from: https://obgyn.onlinelibrary.wiley.com/doi/10.1002/uog.12448
8. Szwast A, Tian Z, McCann M, Soffer D, Rychik J. Comparative analysis of cerebrovascular resistance in fetuses with single-ventricle congenital heart disease. Ultrasound Obstet Gynecol [Internet]. 2012;40:62–7. Available from: https://obgyn.onlinelibrary.wiley.com/doi/10.1002/uog.11147
9. Williams IA, Tarullo AR, Grieve PG, Wilpers A, Vignola EF, Myers MM, et al. Fetal cerebrovascular resistance and neonatal EEG predict 18-month neurodevelopmental outcome in infants with congenital heart disease. Ultrasound Obstet Gynecol [Internet]. 2012;40:304–9. Available from: https://obgyn.onlinelibrary.wiley.com/doi/10.1002/uog.11144
10. Arduini M, Rosati P, Caforio L, Guariglia L, Clerici G, Di Renzo GC, et al. Cerebral blood flow autoregulation and congenital heart disease: possible causes of abnormal prenatal neurologic development. J Matern Neonatal Med [Internet]. 2011;24:1208–11. Available from: http://www.tandfonline.com/doi/full/10.3109/14767058.2010.547961
11. McElhinney DB, Benson CB, Brown DW, Wilkins-Haug LE, Marshall AC, Zaccagnini L, et al. Cerebral Blood Flow Characteristics and Biometry in Fetuses Undergoing Prenatal Intervention for Aortic Stenosis with Evolving Hypoplastic Left Heart Syndrome. Ultrasound Med Biol [Internet]. 2010;36:29–37. Available from: https://linkinghub.elsevier.com/retrieve/pii/S0301562909015312
12. Berg C, Gembruch O, Gembruch U, Geipel A. Doppler indices of the middle cerebral artery in fetuses with cardiac defects theoretically associated with impaired cerebral oxygen delivery in utero : is there a brain-sparing effect? Ultrasound Obstet Gynecol [Internet]. 2009;34:666–72. Available from: https://obgyn.onlinelibrary.wiley.com/doi/10.1002/uog.7474
13. Brossard-Racine M, du Plessis A, Vezina G, Robertson R, Donofrio M, Tworetzky W, et al. Brain Injury in Neonates with Complex Congenital Heart Disease: What Is the Predictive Value of MRI in the Fetal Period? Am J Neuroradiol [Internet]. 2016;37:1338–46. Available from: http://www.ajnr.org/cgi/doi/10.3174/ajnr.A4716
14. Brossard-Racine M, du Plessis AJ, Vezina G, Robertson R, Bulas D, Evangelou IE, et al. Prevalence and Spectrum of In Utero Structural Brain Abnormalities in Fetuses with Complex Congenital Heart Disease. Am J Neuroradiol [Internet]. 2014;35:1593–9. Available from: http://www.ajnr.org/cgi/doi/10.3174/ajnr.A3903
15. Mlczoch E, Brugger P, Ulm B, Novak A, Frantal S, Prayer D, et al. Structural congenital brain disease in congenital heart disease: Results from a fetal MRI program. Eur J Paediatr Neurol [Internet]. 2013;17:153–60. Available from: https://linkinghub.elsevier.com/retrieve/pii/S1090379812001523
16. Schellen C, Ernst S, Gruber GM, Mlczoch E, Weber M, Brugger PC, et al. Fetal MRI detects early alterations of brain development in Tetralogy of Fallot. Am J Obstet Gynecol [Internet]. 2015;213:392.e1-392.e7. Available from: https://linkinghub.elsevier.com/retrieve/pii/S0002937815005220
17. Al Nafisi B, van Amerom JF, Forsey J, Jaeggi E, Grosse-Wortmann L, Yoo S-J, et al. Fetal circulation in left-sided congenital heart

disease measured by cardiovascular magnetic resonance: a case–control study. J Cardiovasc Magn Reson [Internet]. 2013;15:65. Available from: https://linkinghub.elsevier.com/retrieve/pii/S1097664723007810
18. Sun L, Macgowan CK, Sled JG, Yoo S-J, Manlhiot C, Porayette P, et al. Reduced Fetal Cerebral Oxygen Consumption Is Associated With Smaller Brain Size in Fetuses With Congenital Heart Disease. Circulation [Internet]. 2015;131:1313–23. Available from: https://www.ahajournals.org/doi/10.1161/CIRCULATIONAHA.114.013051
19. Limperopoulos C, Tworetzky W, McElhinney DB, Newburger JW, Brown DW, Robertson RL, et al. Brain Volume and Metabolism in Fetuses With Congenital Heart Disease. Circulation [Internet]. 2010;121:26–33. Available from: https://www.ahajournals.org/doi/10.1161/CIRCULATIONAHA.109.865568
20. Masoller N, Sanz-Cortés M, Crispi F, Gómez O, Bennasar M, Egaña-Ugrinovic G, et al. Severity of Fetal Brain Abnormalities in Congenital Heart Disease in Relation to the Main Expected Pattern of in utero Brain Blood Supply. Fetal Diagn Ther [Internet]. 2016;39:269–78. Available from: https://karger.com/article/doi/10.1159/000439527
21. Clouchoux C, du Plessis AJ, Bouyssi-Kobar M, Tworetzky W, McElhinney DB, Brown DW, et al. Delayed Cortical Development in Fetuses with Complex Congenital Heart Disease. Cereb Cortex [Internet]. 2013;23:2932–43. Available from: https://academic.oup.com/cercor/article-lookup/doi/10.1093/cercor/bhs281
